# Analysis of the effects of workplace physical activity in companies—a literature review

**DOI:** 10.47626/1679-4435-2022-660

**Published:** 2023-02-03

**Authors:** Luana dos Santos Viana, Taciane Machado de Melo Pereira

**Affiliations:** 1 Faculdade de Integração do Sertão, Serra Talhada, PE, Brazil.

**Keywords:** quality of life, physical activity, physical therapy, absenteeism, RSI/WRMSD, qualidade de vida, atividade física, fsioterapia, absenteísmo, LER/DORT

## Abstract

Quality of work life is understood to be directly linked to the level of satisfaction of an individual with the execution of his or her tasks. Workplace physical activity is an important activity that aims to relax the muscle groups most used in occupational tasks, to increase workers’ enthusiasm, and to reduce sickness absenteeism, thus contributing to improvements in quality of life. This study aimed to analyze the effects of the implantation of workplace physical activity protocols at companies. We performed a literature review in the LILACS, SciELO, and Google Scholar databases using the following keywords: “quality of life,” “exercise therapy,” and “occupational health.” With this search, we obtained 73 studies, of which 24 were selected afer reading the titles and abstracts. Afer full reading of the studies and applying eligibility criteria, 16 articles were excluded and the remaining eight were used in this review. By analyzing these eight studies, we were able to verify the benefits of workplace physical activity in improving quality of life, reducing pain intensity and frequency, and preventing occupational diseases. Workplace physical activity programs, when performed at least three times a week, provide various benefits to workers’ health and wellbeing, especially in the reduction of aches and pains and musculoskeletal discomfort, which directly influences improvements in quality of life.

## Abstract (pt)

Compreende-se que a qualidade de vida no trabalho está diretamente ligada ao nível de satisfação do indivíduo em relação à execução de suas tarefas. A ginástica laboral é uma atividade física importante que tem como objetivos relaxar os grupos musculares mais requisitados durante a execução das tarefas ocupacionais, proporcionar o aumento no entusiasmo dos trabalhadores e reduzir o absenteísmo por motivos de saúde, contribuindo, assim, com a melhora da qualidade de vida. Este estudo objetivou analisar os efeitos da implantação de protocolos de ginástica laboral nas empresas. Foi realizado um levantamento bibliográfico nas bases de dados LILACS, SciELO e Google Acadêmico. Foram utilizadas as palavras-chaves “qualidade de vida”, “terapia por exercício” e “saúde do trabalhador”. Após a pesquisa, foram obtidos 73 artigos, dos quais 24 foram selecionados com base na leitura de títulos e resumos. Após a leitura integral e aplicação dos critérios de elegibilidade, foram excluídos 16 artigos, restando oito para a elaboração do estudo. Através da análise dos oito artigos, foi possível observar os benefícios advindos da prática da ginástica laboral na melhoria da qualidade de vida, redução da intensidade e frequência da dor e prevenção de doenças ocupacionais. Os programas de ginástica laboral, quando desempenhados numa periodicidade de no mínimo três sessões semanais, proporcionam diversos benefícios no que tange à saúde e ao bem-estar dos trabalhadores, especialmente na redução de quadros álgicos e desconfortos osteomusculares, o que influencia diretamente na melhora da qualidade de vida.

## INTRODUCTION

Quality of work life (QWL) is understood to be directly linked to an individual’s level of satisfaction with the execution of his or her tasks, where satisfaction and wellbeing are indispensable aspects for the good functioning of a company. Numerous factors afect quality of life (QoL), among which we highlight as aspects interfering with QWL the environmental, family, health, and leisure conditions, as well as working conditions, since most of the active population spends part of their time in this environment.^1^

Amorim^2^ states that taking aspects related to workers’ physical and mental health into consideration directly resonates with a company’s level of productivity and success. This reinforces the importance of the implementation of QWL programs by employers, aiming to broaden employees’ participation, satisfaction, and health, and thus obtaining increased productivity and profits for the company.

Costa & Russos^3^ explain that occupational hazards that afect workers result from physical, chemical, psychosocial, ergonomic, and/or biological factors. Hazards resulting from noises, vibration, and temperature are considered as physical, whereas those caused by bacteria, fungi, parasites, or viruses are considered biological hazards. Chemical hazards are those able to enter the body through the respiratory route in the form of dust, smoke, gas, or similar compounds, or via skin absorption or ingestion. Fatigue and tension are related to psychosocial hazards, which are usually associated with the number of hours worked, overtime, lack of control over work, among others. Ergonomic hazards are directly linked to postures adopted during working hours, non-adjustable furniture, and inadequate working conditions.^3^

Among the consequences of exposure to ergonomic risk factors, we highlight the appearance of repetitive strain injuries (RSI) and work-related musculoskeletal disorders (WRMSD), which originate in or are aggravated by repetitive movements, static work, an excessive work pace, and/or unhealthy biomechanics in the workplace.^4^

RSI/WRMSD represent a set of diseases characterized by some clinical manifestations with various degrees of intensity, such as myalgia and nerve and tendon injuries. The etiology of this set of disorders is complex and frequently produced by repetitive movements required for the execution of a professional task or even by the adoption of certain postures during long periods; both of these factors lead to an overload of the muscles and adjacent structures. This picture can even be intensified if it happens alongside job dissatisfaction, which puts psychosocial factors also as predisposing to RSI/WRMSDs.^5^,^6^

Preventing or minimizing the overload caused by work activities on the musculoskeletal system is an attribution of occupational physical therapists. This professional should act by employing ergonomics, investigating the mechanisms of pain and discomfort presented by employees, and giving lectures to raise awareness on the importance of preventing work-related diseases. The physical therapist is also prepared to develop workplace physical activity (WPA) programs that meet the workers’ needs and fit their occupations.^7^

WPA is an important physical activity performed at the workplace that aims, among other things, to relax the muscle groups most used in occupational tasks. It consists in sessions that last around 10 to 15 minutes and include global stretches, strengthening of target muscle groups, exercises for improving motor coordination, and relaxation activities. It is worth mentioning that WPA not only acts therapeutically in musculoskeletal disorders, but also in a preventive fashion.^8^

For understanding the particularities of WPA, it is important to note that it is subdivided into three types. Preparatory WPA, as the name suggests, is performed at the beginning of the workday, whereas compensatory WPA occurs exactly where the employee performs his or her activities, during work hours. Last, but not least, relaxation WPA occurs at the end of the workday and generally aims to eliminate tensions accumulated during the occupational activity.^9^ Soares et al.^10^ highlight that WPA increases the enthusiasm of workers with their occupational activities, in addition to reducing sickness absenteeism, thus contributing to an improved QoL when performed correctly and regularly.

Considering the important role of WPA within the workplace, this study aimed to analyze the effects of the implantation of WPA protocols in companies.

## METHODS

This is an integrative literature review. We initially performed an electronic literature search between May and June 2020, considering studies indexed in the Latin American and Caribbean Health Sciences Literature (LILACS), Scientific Electronic Library Online (SciELO), and Google Scholar databases. The search used descriptors related to the main subject and the focus of the study: “quality of life,” “physical activity,” and “occupational health.”

Subsequently, the relevant studies were carefully selected through the following steps: title screening, abstract reading, and full-text analysis. The inclusion criteria used in this review were: free original articles, in Portuguese, fully available in the databases, on WPA and its effects and risks, as well as working conditions and QoL at companies, published between 2010 and 2020. The exclusion criteria considered studies that did not consider the selected theme, were not in Portuguese, and were not experimental.

**Figure 1:**
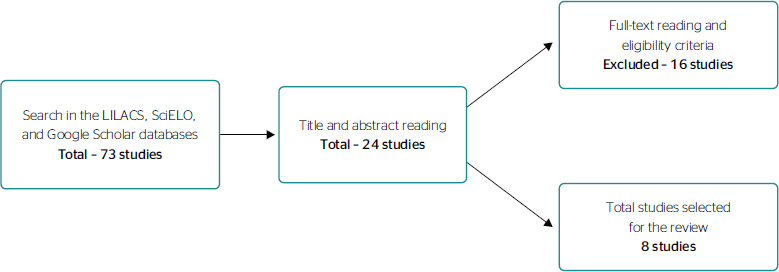
Stages of article selection, according to the established criteria, in the Latin American and Caribbean Health Sciences Literature (LILACS), Scientific Electronic Library Online (SciELO), and Google Scholar databases, 2020.

Figure 1 represents the stages of the careful selection of the eight studies that comprised the present review. Considering ethical aspects, the data published by authors of the selected studies were fully respected and maintained.

## RESULTS

Table 1 shows an overview of the selected articles, with information on author names, title, objective, and conclusion for each of the mentioned articles.

**Table 1:** Distribution of studies according to author name/year, title, objectives, and conclusion

Author	Title	Objective	Sample	Conclusion
Candotti et al.^11^	Labor Gymnastic’s effects on low back pain and postural habits adopted in the working environment	To verify the effect of WPA on back pain and posture habits of workers who remain seated for long periods.	30	The study concluded that WPA presented positive results as to pain reduction, mainly in the back region, and improvements in posture habits.
Brito & Martins^16^	Perceptions of the participants of a WPA program on flexibility and factors related with a healthy lifestyle.	To verify the perception of workers on trunk and hip flexibility, in addition to other factors related with a physically active and healthy lifestyle after adhering to a WPA program at Universidade da Paraíba.	10	Numerous benefits were verified with the application of a WPA program, such as improved flexibility of the analyzed regions, reductions in musculoskeletal pain, and increased disposition for work and social interaction.
Machado Junior et al.^14^	Musculoskeletal complaints and labor gymnastic practice of financial institution employees	To identify the musculoskeletal complaints of 16 workers of a financial institution, who practiced WPA or not, through a body map questionnaire.	16	Considering the low frequency of WPA (twice a week), researchers were not able to diferentiate the intensity of complaints, which indicated a need to adjust activities, aiming at a higher effectiveness.
Grande et al.^15^	Comparison of worker’s health promotion interventions: a cluster randomized controlled trial	To compare different occupational health promotion interventions and their impact on QoL domains (health, physical activity, occupational environment, and perception of QoL).	172	The working environment of the companies where WPA was applied was significantly improved.
Dartora & Santos^13^	Preparatory workplace kinesiotherapy for urban cleaning workers of a company in the Taquari valley/RS.	To analyze and intervene in the health of urban cleaning workers of a small-sized company in the Taquari valley, minimizing risks of musculoskeletal injuries.	8	Workplace kinesiotherapy and guidance had a positive impact on the daily lives of workers during work hours.
Freitas et al.^17^	The effects of compensatory workplace exercises to reduce work-related stress and musculoskeletal pain	To evaluate the effect of a compensatory WPA program on workers, aiming at reducing occupational stress and musculoskeletal pain.	30	Researchers concluded that compensatory WPA reduced musculoskeletal pain in most body segments. The improvement was considered statistically significant in virtually all segments, except for the upper limb. Occupational stress complaints were not reduced.
Beneli & Acosta^12^	Efects of a workplace physical activity program on the incidence of pain among workers of a software company.	To analyze the incidence of pain among workers of a company in the informatics sector and to evaluate the effects of a WPA program.	21	Researchers concluded that WPA performed three times a week for 6 months could significantly contribute to reducing or eliminating pain among employees.
Nascimento et al.^8^	Benefits of workplace kinesiotherapy in employees of the administration sector.	To evaluate the benefits of workplace kinesiotherapy among employees of the administration sector with a workplace physical activity intervention program.	21	Researchers proved that a workplace kinesiotherapy program brought important benefits to workers’ health and QoL, in addition to preventing the appearance of WRMSD.

WRMSD = work-related musculoskeletal disorders; WPA = workplace physical activity; WPAP = workplace physical activity program; QoL = quality of life.

## DISCUSSION

Technological and organizational innovations are on the rise in the workplace, thus demanding a greater adaptation of workers to new technologies. These adjustments frequently end up happening at a fast pace and in association with long working hours, which has led to musculoskeletal discomfort and increased musculoskeletal injuries.^11^

According to Beneli & Acosta,^12^ WPA results in numerous benefits to employees and, consequently, to the company. Alleviating pain and discomfort due to occupational activities is among the benefits most cited in the literature. In a study performed by Candoti et al.^11^ with a sample of 30 employees divided into control and experimental groups, a decrease in back pain intensity and frequency was observed afer 3 months of a WPA protocol applied three times a week (as reported by workers in the experimental group), in addition to improvements in their posture habits. Corroborating this study, Dartora & Santos^13^ evaluated the application of preparatory WPA in urban cleaning employees with a frequency of three sessions a week for 2 months and observed a reduction in the aches and pains reported by these workers, as well as greater disposition for performing work tasks.

Machado Jr et al.,^14^ on the other hand, aimed to identify musculoskeletal complaints in individuals who practiced WPA or not, considering a sample of 16 employees. Tose who participated in WPA presented milder complaints, only in the neck region, when compared to the group who did not participate. The authors justify the result significance only in this region because of the frequency of WPA, which was performed only twice a week. Based on the data presented above, we note the importance of the number of weekly sessions, since most of the studies with positive results indicate a minimum frequency of three sessions a week.

The occupational QoL domains were analyzed by Grande et al.^15^ in a cluster randomized controlled study, where different health promotion interventions were applied to various groups. The promotion of health awareness through lectures and a WPA protocol for three months were among the procedures adopted in this study. The obtained results led to the conclusion that a significant improvement of the working environment happened where WPAwas applied; however, when considering the health and perception of QoL domains, three months were insuficient for reporting significant improvements.

Difering slightly from the study by Grande et al.,^15^ Brito & Martins^16^ evaluated the perception of QoL of 10 workers and presented data that favor different aspects of QoL, referring to the health of the studied employees. Nascimento et al.^8^ applied a WPA protocol and also obtained positive results regarding occupational health and QoL.

One of the great impacts of work activities, especially when performed in a static position, is decreased flexibility, mainly of the posterior chain muscles. Based on this viewpoint, Brito & Martins^16^ performed a study with the aim of verifying the perception of workers on trunk and hip flexibility, in addition to factors related with a physically active and healthy lifestyle. Ten employees were evaluated and underwent a WPA protocol, and a positive repercussion was identified in the lifestyle and QoL of participants, since improved flexibility of the analyzed regions, reductions in musculoskeletal pain, and increased disposition for work and social interaction were among the reported benefits.

Flexibility was also one of the study objectives of Nascimento et al.,^8^ where 21 employees were evaluated and underwent a WPA protocol for 6 months, with three sessions a week. The authors obtained statistically significant improvements in hamstring muscle flexibility, in addition to findings related to reduced musculoskeletal pains and aches and increased QoL among these workers.

When observing the type of WPA more frequently applied in protocols with positive results, we observe a higher predominance of studies that chose to use compensatory physical activity as a tool.^8^,^16^,^17^ This can be justified by the fact that this type of WPA allows a larger time window for executing sessions, since it can be performed at any moment of working hours, in addition to being widely used in administrative environments, which are the main focus of the selected studies.

Freitas et al.^17^ performed a study with 30 employees, aiming to evaluate the effect of a compensatory WPA program in reducing complaints related to occupational stress and musculoskeletal pain. Pain reduction was considered statistically significant in virtually all body segments, except for the upper limb. However, when it comes to occupational stress, no relevant reduction was statistically detected, and the authors associated this result with a reduced sample size.

The current occupational scenario, made up of an intense workday and increased musculoskeletal symptoms, highlights the need for investments directed at the physical and mental wellbeing of these workers. In addition to investing in QoL and improving workplace conditions, physical activity performed in this environment is known to provide employees with moments of leisure and relaxation, minimizing the occurrence of occupational disorders and leading to increases in productivity and quality of the service provided by employees.^12^

## CONCLUSIONS

The results suggest that WPA programs, when performed at least three times a week, provide various benefits to workers’ health and wellbeing, especially in the reduction of aches and pains and musculoskeletal discomfort, in addition to a general improvement of QWL. Given these results, we highlight the importance of inserting a physical therapist in the workplace, since interventions by this professional promote improvements in the physical, mental, and social wellbeing of workers, which results in a reduced incidence of occupational diseases, as well as increased productivity and, consequently, increased company success. Nevertheless, more studies are suggested on this subject, with larger samples and longer intervention times to better confirm the results of this review.
